# Perceived transformational leadership from the coach and athletes’ subjective well-being: A moderated mediated model

**DOI:** 10.3389/fpsyg.2022.1100645

**Published:** 2023-01-16

**Authors:** Wenhao Liu, Wenzhuang Wang, Shangjian Yang

**Affiliations:** ^1^College of Physical Education, Shandong University, Jinan, China; ^2^School of Health Care Security, Shandong First Medical University, Jinan, China

**Keywords:** transformational leadership, basic psychological needs, well-being, coach, athlete

## Abstract

Based on human motivation theory and self-determination theory, this study investigated the impact of coaches’ transformational leadership on athletes’ well-being, and the influences of gender and athletes’ basic psychological needs. The survey objects comprised of 432 athletes participating in the Hebei Games in China. The results showed that coaches’ transformational leadership could significantly and positively predict athletes’ well-being. Basic psychological needs had a mediating effect on the relationship between coaches’ transformational leadership and athletes’ well-being. Gender significantly moderated the effect between athletes’ basic psychological needs satisfaction and subjective well-being. Compared with male athletes, female athletes had their basic psychological needs met, and the improvement in their well-being was more significant than the males. Coaches should adapt their transformational leadership to directly improve athletes’ well-being and indirectly achieve their well-being by satisfying the athletes’ basic psychological needs.

## 1. Introduction

Competitive sports are highly practiced social activities (Li, 2019). In a sports training team, athletes constitute the main body of the sports training activities, bear the sports training load, and reveal the sports training results. The coaches are responsible for the sports training plan and organize and instruct the sports training activities. Therefore, coaches and athletes have a complex social relationship (Guo and Hu, 2011). Due to different national conditions, systems, and mechanisms, Chinese competitive sports coaches’ roles and statuses may be higher than those in other countries (Chen and Chen, 2021), as under the influence of traditional Chinese culture, coaches convey paternalistic leadership. This kind of leadership often leads to a contradiction between athletes and coaches because of the strict management of athletes, thus reducing athletes’ well-being (Gao et al., 2013; Yang et al., 2020). Therefore, to fully enhance athletes’ well-being, it is necessary to actively explore new ways of leadership to sustainably develop competitive sports. The coaches place higher leadership ability requirements on the athletes in an attempt to show more robust adaptability and higher flexibility within the new leadership modes. New leadership theory believes that transformational leadership can meet such needs. Therefore, it is of academic and real-world value to explore the influence and mechanisms of coaches’ transformational leadership on athletes’ well-being (Price and Weiss, 2013).

Transformational leadership was initially proposed by Burns (1978, 2004) and then further developed by other authors for application in the industry, military, educational, and sporting fields (see, for example, Bass and Avolio, 1994; Bass, 1998; Arthur et al., 2017). Transformational leadership allows employees to realize the significance of a task, inspires motivation, and stimulates employees’ self-implementation, self-monitoring, and self-control (Bass, 1999). Transformational leadership builds mutual trust and an organizational atmosphere, encouraging employees to sacrifice their interests and go beyond the desired outcomes to achieve collaborative successful (Bass, 1999). In recent years, the transformational leadership concept has been gradually developed in sports. Early scholars worldwide have adopted Bass’s concept but have described the “leader” as the “coach” and replaced the “employees” with the “athletes” (Charbonneau et al., 2001; Yan et al., 2017). However, Callow et al. (2009) point out that the sports field differs from the business field as in the transformational leadership context, coaches should set examples and play exemplary roles. Moreover, coaches should provide personalized care for athletes and encourage them to both unlock their potential and exhibit a high level of competitiveness (Callow et al., 2009). Simultaneously, athletes are encouraged to have a high sense of identity within the team but should place the team’s interests above their own. Finally, athletes who perform well should be appropriately rewarded (Callow et al., 2009). Sports scholars have widely recognized this perspective (Hardy et al., 2010; Arthur et al., 2011; Vella et al., 2012; Smith et al., 2017). In the current study, coaches’ transformational leadership refers to athletes’ perceived transformational leadership behavior of coaches.

Subjective well-being refers to an individual’s general, comprehensive, and long-term satisfaction with their situation (Eid and Diener, 2004). It can be affected by many factors, such as cognition, occupation, and society (Diener, 2000). Emotion is one of the most critical factors for determining well-being (Li, 2014). Athletes form a particular group under the “nationwide system” and experience a relatively solitary environment (Sun and Yang, 2013). Their emotional well-being primarily comes from their interactions with coaches and teammates, and the coaches play a significant role (Sun and Yang, 2013). A survey of Chinese athletes reveals that coaches’ leadership styles are essential indicators of athletes’ well-being (Jiang et al., 2013); Sterling and Tafvelin (2014) further confirm that coaches’ transformational leadership significantly impacts athletes’ well-being. However, in what ways do coaches’ transformational leadership styles affect athletes’ well-being?

Building from the human motivation theory (McClelland and Mac Clelland, 1967), Deci and Ryan’s (2008) self-determination theory identifies three basic psychological needs that motivate human behavior and well-being: autonomy, competence, and relatedness. Bass (1999) asserts that coaches often use motivation and implement a shared vision to satisfy athletes’ independent needs. Moreover, coaches use intelligence stimulation and set high-performance expectations to meet athletes’ ability needs (Bass, 1999). Finally, the athletes’ belonging needs are met through rewards (Bass and Avolio, 1990); simultaneously, coaches can satisfy athletes’ basic psychological needs by creating an excellent environment and atmosphere (Bean et al., 2020). Overall, regulating athletes’ basic psychological needs can enhance their psychological toughness and athletic performance (Yang et al., 2020).

Further, satisfying athletes’ basic psychological needs may improve their well-being (Liu and Zhang, 2010). According to self-determination theory (Deci and Ryan, 2008), basic psychological needs are the main factors for developing well-being and physical and mental health (Liu and Zhang, 2010). They are also key to internalizing intrinsic and extrinsic motivations and guide the direction and sustainability (Liu and Zhang, 2010). Meanwhile, meeting basic psychological needs directly improves well-being (Deci and Ryan, 2000); this has been supported by empirical evidence in the sports research. For example, a study on teenage footballers and cricketers in England has shown that coaching behavior that meets the athletes’ basic psychological needs can predict their well-being (Reinboth et al., 2004); this has been further verified by a study on football players in England (Adie et al., 2012). Based on the above, this study posits that:

*H1*: Basic psychological needs mediates the relationship between coaches’ transformational leadership and athletes’ well-being.

The mediating effect of athletes’ basic psychological needs and the influence of coaches’ transformational leadership on athletes’ well-being may be regulated by gender, as follows. First, the degree of well-being between male and female athletes differs (Fraser, 2012), as female athletes have higher levels of well-being than males (Wang et al., 2008; Liu, 2011). Second, the psychological research has revealed differences in the satisfaction of basic psychological needs between males and females (Kashdan et al., 2009). Thus, gender regulates the relationship between basic psychological needs and well-being (Gómez-Baya et al., 2018). The sports research has shown that male and female athletes have different basic psychological needs (Bussey and Bandura, 1999; Adie et al., 2008; Jordalen and Lemyre, 2014); however, whether this moderates athletes’ well-being requires further testing.

Finally, self-determination theory asserts that males prefer the information selection process than the information collection process, as it is more independent and purposeful. Meanwhile, females are more inclined to collect more comprehensive information and need more emotional support to acquire the information (Wigfield et al., 2015). Conversely, human motivation theory states that males use communication to obtain information and achieve a specific purpose (Cai, 2016). Female communication is based on relationship maintenance and emotional communication and focuses more on satisfying psychological needs (Cai, 2016). Therefore, male and female athletes have different psychological information exchanges and emotional communication styles with their coaches (Cai, 2016). Based on the above, this study posits that:

*H2*: The direct impact of coaches’ transformational leadership on athletes’ well-being and its mediating process is moderated by gender.

## 2. Methods

### 2.1. Participants and procedure

The study sample comprised of 36 teams participating in the final stages of football, basketball, and volleyball at the Hebei Games, totaling 432 athletes (227 males, 205 females), with an average age of 17.31 ± 1.32 years. Data were collected *via* a questionnaire survey. With the help of the referees and coaches, the research team conducted the questionnaire survey at the athletes’ residences during their downtime. The survey period lasted for 22 days. A total of 432 questionnaires were distributed and collected (recovery rate = 100%). After eliminating 12 invalid questionnaires, 420 valid questionnaires were obtained (response rate = 97.22%).

This research was reviewed by the Ethics Committee of Shandong First Medical University (No. R202202220014) and was supported and authorized by the Ethics Committee of Shandong First Medical University. All adult participants provided their informed consent, and all minors’ consent was obtained from their parents or legal guardians prior to participating. The study was conducted in line with the Declaration of Helsinki.

### 2.2. Measures

This study used the Differentiated Transformational Leadership Inventory (DTLI: Arthur et al., 2011) to measure coaches’ transformational leadership. The DTLI included 7 dimensions: individualized treatment, motivational stimulation, intellectual inspiration, cultivation and acceptance of organizational goals, high-performance expectation, virtue norms, and contingency rewards, with a total of 27 items. The items were rated on a 5-point Likert scale.

This study used the Athletes’ Basic Psychological Needs Scale (ABPNS: Cai, 2016) to assess athletes’ basic psychological needs. The scale included three dimensions: perceived competence, perceived autonomy, and perceived belonging, with a total of 15 items, and Cronbach’s alphas of the dimensions were 0.87, 0.87, and 0.86, respectively (Cai, 2016). The items were rated on a 5-point Likert scale.

This study adopted the Subjective Well-Being of Chinese Athletes (SWCA: Jiang et al., 2014) questionnaire to measure athletes’ well-being. The questionnaire contained 7 dimensions, including training competition satisfaction, interpersonal satisfaction, positive emotion, social security satisfaction, living environment satisfaction, negative emotion, and self-development satisfaction, with a total of 38 items. Cronbach’s alpha was between 0.58 and 0.85 (Jiang et al., 2014). The items were rated on a 5-point Likert scale.

### 2.3. Data analyses

This study used SPSS 25.0 for the descriptive statistical. This study also applied AMOS Graphics for CFAs of every questionnaire. Again SPSS for Cronbach’s alpha analysis, and preliminary analyses. At last, SPSS macro extension “Process” (Hayes, 2015) was utilized for testing the mediated moderated model.

Due to the athletes’ self-reported data, there may have been common method bias. Therefore, this study used program control and statistical tests to test for possible common method bias. In the program control, anonymity, forward, and reverse scoring tests, this study separately investigated the antecedent and result variables to control the system *via* Harman’s single factor test. The results showed that the first factor explained 23.16% of the total variance without rotation, which was far below the standard of 40%. Therefore, the data had no common method bias.

## 3. Results

### 3.1. Descriptive statistics and preliminary analyses

In this study, Cronbach’s alpha of DTLI was 0.92. The fitting indexes were as follows: *χ*^2^/*df* = 2.01, GFI = 0.91, AGFI = 0.91, RMR = 0.06, RMSEA = 0.03, NFI = 0.90, RFI = 0.92, and IFI = 0.97. Similarly, Cronbach’s alpha of ABPNS was 0.92. The fitting indexes were as follows: *χ*^2^/*df* = 2.26, GFI = 0.93, AGFI = 0.90, RMR = 0.05, RMSEA = 0.05, NFI = 0.92, RFI = 0.93, and IFI = 0.92. Finally, the Colonbach coefficient of the third scale SWCA was 0.96. The fitting indexes were as follows: *χ*^2^/*df* = 3.11, GFI = 0.90, AGFI = 0.89, RMR = 0.06, RMSEA = 0.02, NFI = 0.91, RFI = 0.90, and IFI = 0.91.

This study compared the perceived transformational leadership of coaches, basic psychological needs, and well-being of male and female athletes. The independent sample *t*-test results showed no significant difference (*t* = −0.49, *p* > 0.05) for perceived transformational leadership of coaches between male athletes’ (M = 4.09, SD = 0.71) and female athletes (M = 4.18, SD = 0.58). The basic psychological needs score of male athletes (M = 3.12, SD = 0.81) was lower than that of female athletes (M = 3.91, SD = 0.69), and the difference was significant (*t* = −7.38, *p* < 0.001). The well-being of male athletes (M = 3.52, SD = 0.52) was lower than that of female athletes (M = 4.34, SD = 0.31), and the difference was significant (*t* = −7.56, *p* < 0.001). This study conducted a correlation analysis for each variable, and the results showed significant positive correlations among all variables (Table 1).

**Table 1:** Descriptive statistics and correlations among the variables.

	M	SD	1	2	3
Coaches’ transformational leadership	4.12	0.68	1		
Athletes’ basic psychological needs	3.45	0.72	0.311^***^	1	
Athletes’ well-being	4.11	0.44	0.444^***^	0.456^***^	1

*** Indicates *p* < 0.001.

### 3.2. Main analyses

**Figure 1:**
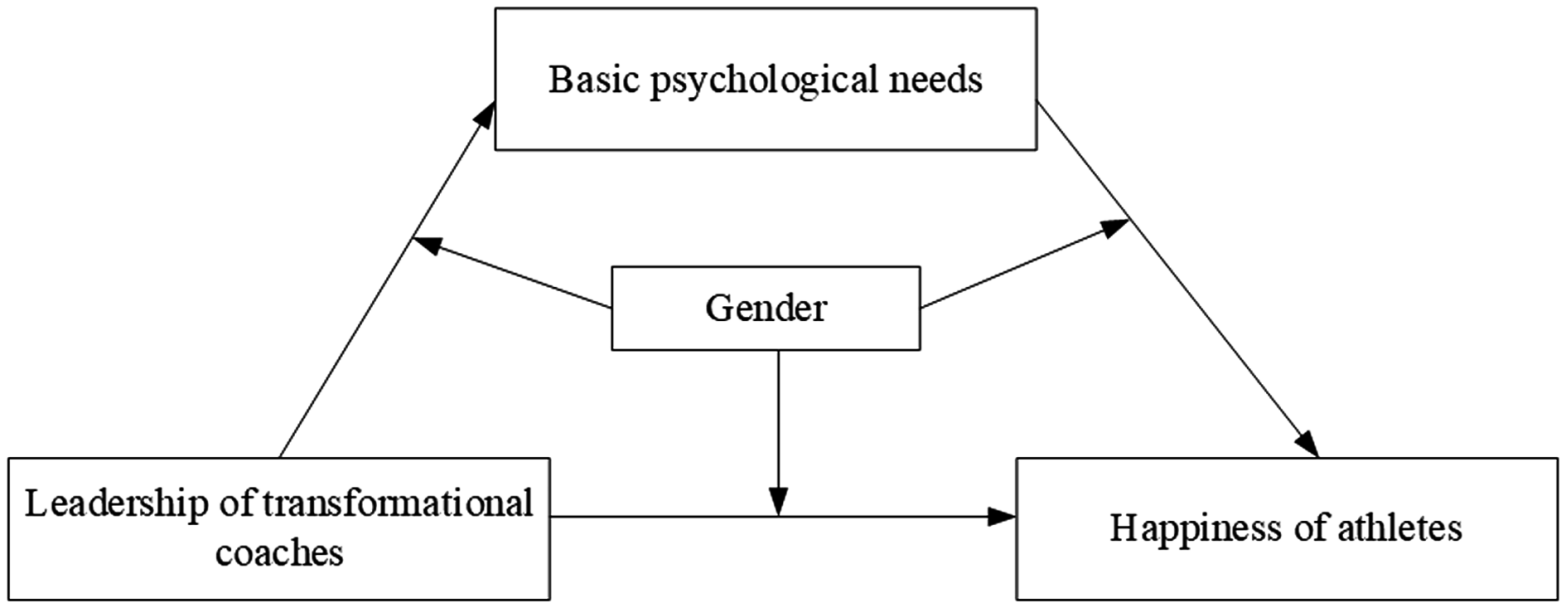
Transformational leadership, basic psychological needs, gender, and well-being hypothesis model.

This study used Wen and Ye’s (2014a,b) method to test the coefficients of the three regression equations (Bolin, 2014). Equation 1 tested whether the direct effect of coaches’ transformational leadership on athletes’ well-being was regulated by gender. Equation 2 tested whether the influence of coaches’ transformational leadership on athletes’ basic psychological needs was regulated by gender. Equation 3 tested whether the impact of athletes’ basic psychological needs on their well-being was regulated by gender. The gender variables were virtualized and the other variables were standardized. The parameter estimation of Equation 2 is shown in Table 2, and the parameter estimations of Equations 1 and 3 are shown in Table 3. This study used the SPSS Process macro (Hayes, 2015) for testing the model depicted in Figure 1.

**Table 2:** Predictive effects of transformational leadership and gender on athletes’ basic psychological needs.

Predictive variables	*B*	*SE*	*T*	*p*	95% CI
Coaches’ transformational leadership	0.51	0.06	9.97	<0.001	[0.33, 0.49]
Gender	−0.55	0.07	−10.48	<0.001	[−0.63, −0.39]
Coaches’ transformational leadership*gender	−0.09	0.05	−3.24	>0.05	[−0.23, 0.06]

The coefficients in the table are non-standardized.

**Table 3:** Predictive effects of transformational leadership, basic psychological needs, and gender on athletes’ well-being.

Predictive variables	*B*	*SE*	*T*	*p*	95% CI
Coaches’ transformational leadership	1.01	0.21	6.77	<0.001	[0.65, 1.39]
Gender	−1.75	0.37	−8.47	<0.001	[−2.68, −1.31]
Coaches’ transformational leadership*gender	0.29	0.25	0.99	>0.05	[−0.10, 0.78]
Athletes’ basic psychological needs	3.56	0.37	16.96	<0.001	[3.56, 4.29]
Athletes’ basic psychological needs*gender	−1.54	0.29	−7.68	<0.001	[−2.11, −0.98]

The coefficients in the table are non-standardized.

Table 2 shows that coaches’ transformational leadership has a significant positive predictive effect on athletes’ basic psychological needs. Gender has a significant negative predictive effect on athletes’ basic psychological needs. However, the interaction between coaches’ transformational leadership and gender has no significant effect on athletes’ basic psychological needs. These results indicate that gender does not play a moderating role in the relationship between coaches’ transformational leadership and athletes’ basic psychological needs.

Table 3 shows that coaches’ transformational leadership has a significant positive predictive effect on athletes’ well-being. Gender has a significant negative predictive effect on athletes’ well-being. However, the interaction between coaches’ transformational leadership and gender is not significant. These results indicate that gender does not play a moderating role in the relationship between coaches’ transformational leadership and athletes’ well-being. Basic psychological needs has a significant positive predictive effect on athletes’ well-being. The interaction between basic psychological needs and gender has a significant impact on athletes’ well-being, indicating that gender plays a moderating role in the relationship between athletes’ basic psychological needs and well-being.

Further, the moderating effect of gender is only significant in the latter half of the relationship path between coaches’ transformational leadership, athletes’ basic psychological needs, and athletes’ well-being; thus, the model is a moderated mediation model. According to Hayes (2015), the index should first be determined to assess whether a moderated mediating effect exists. This study’s analysis showed an index of −0.51 and a 95% confidence interval of the Bootstrap test (−0.87, −0.21). The interval did not contain 0, indicating that the model had a significant moderated mediating effect (Hayes, 2015).

This study assigned gender as male (1) and female (0). This study then analyzed the different mediating effects on male and female athletes (Table 4). The results showed that the indirect effect of coaches’ transformational leadership on male athletes’ well-being was significant through the mediation of basic psychological needs, with an effect value of 0.31, which accounted for 27.36% of the total effect. The indirect effect of coaches’ transformational leadership on female athletes’ well-being through the mediation of basic psychological needs was also significant, with an effect value of 0.73, which accounted for 49.15% of the total effect. Therefore, H1 is validated.

**Table 4:** Mediating effect of gender on athletes’ basic psychological needs.

Mediating variables	Gender	Indirect effect value	Boot standard error	Boot LLCI	Boot ULCI
Athletes’ basic psychological needs	Male	0.31	0.07	0.33	0.51	Female	0.73	0.07	0.53	1.11

**Figure 2:**
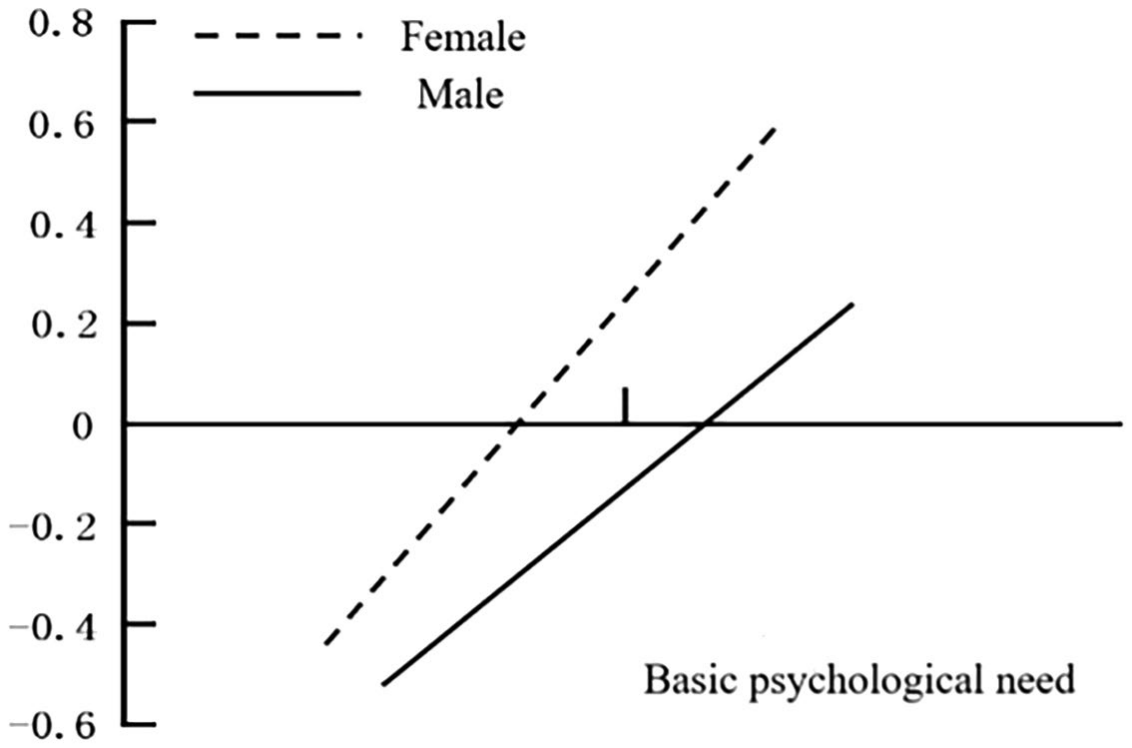
Moderating effect of gender on the relationship between athletes’ basic psychological needs and well-being.

**Figure 3:**
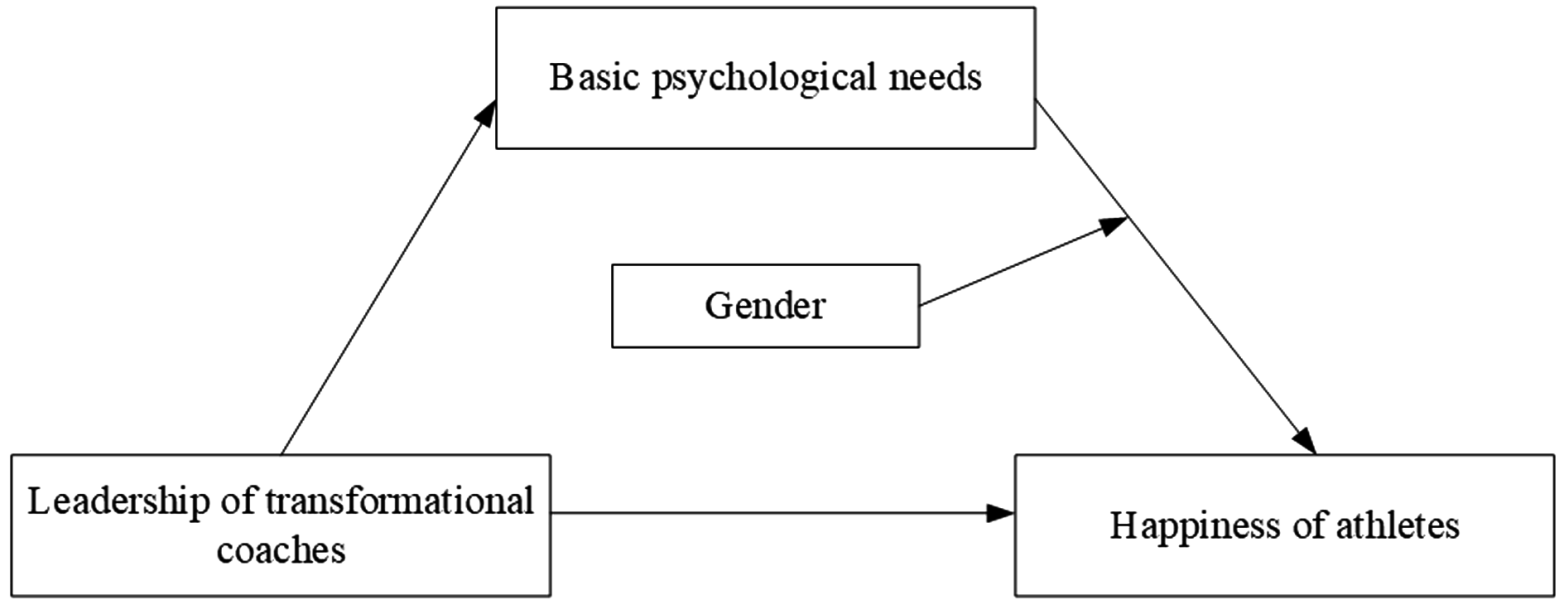
Final model of this study.

This study further assessed the moderating effect of gender in the model *via* a slope test (Figure 2). The results show that basic psychological needs have a higher predictive effect on the well-being of female athletes (simple slope = 0.53, *t* = 16.26, *p* < 0.001) than of male athletes (simple slope = 0.35, *t* = 8.25, *p* < 0.001). These results indicate that when athletes’ basic psychological needs are met, female athletes are more likely to have higher improvements in their well-being than male athletes. Therefore, H2 is partially validated. Figure 3 shows the verification results of Figure 1.

## 4. Discussion

### 4.1. Direct effects of coaches’ transformational leadership and athletes’ basic psychological needs on athletes’ well-being

With the increase in the research on the influence and mechanisms of transformational leadership on the well-being of subordinates in the business field, this study extends the connotation of transformational leadership from the business field to the sports field, which is a positive transfer (Kovach, 2018). The transformational leadership concept may be more applicable to team projects that pursue teamwork and interpersonal communication, such as football, basketball, and volleyball. Studies have shown that coaches play a central and critical role in influencing athletes’ psychology and behavior in sports teams (Davis and Jowett, 2014). Compared with transactional leadership, coaches’ transformational leadership can better improve leadership effectiveness (Rowold, 2006; Xie and Wu, 2017). The current study’s results indicate that coaches’ transformational leadership affects athletes’ well-being, which is consistent with the business research findings (Xie and Wu, 2017; Hu, 2018). Alike business teams, coaches of collective sports teams lead team goals, encourage team morale, enhance team cohesion, and activate players’ expression through transformational leadership to improve athletes’ well-being (Sterling and Tafvelin, 2014). Moreover, according to the theory of well-being, the primary source of people’s subjective well-being is the realization of goals, and one of the connotations of achieving goals is through motivation (Miao and Yu, 2003). Therefore, the current study’s results further support this viewpoint on well-being.

This study also shows that athletes’ basic psychological needs directly impact their well-being, which is consistent with the research on the predictive effects of different groups’ basic psychological needs on their well-being (Ye et al., 2015; Luo and Mu, 2017). Conversely, other studies have verified the promoting effect of basic psychological needs on well-being through experimental intervention (Liu, 2018). According to self-determination theory, autonomy, competence, and relatedness constitute the three basic psychological needs of human beings, and any of these can independently promote well-being. These basic psychological needs exist in different group activities. If individuals can satisfy these psychological needs from their environment, then they may achieve individual growth and become happier (Reinboth et al., 2004). Meanwhile, football, basketball, and volleyball are sports that require strong interpersonal group interactions (Novitaria and Subarkah, 2018; Yu et al., 2018). If athletes undergo collective training as a result of the coaches’ leadership and influence, then they will experience a harmonious team atmosphere and environment, and their well-being will be enhanced.

### 4.2. Mediating role of basic psychological needs

In addition to the direct impact on well-being, this study also shows that coaches’ transformational leadership can indirectly impact athletes’ well-being by meeting their basic psychological needs. Moreover, coaches’ transformational leadership can improve athletes’ perceived competence, self-reliance, and sense of belonging to enhance their well-being. For football, basketball, volleyball, and other collective sports teams, cohesion is a significant index essential for meeting athletes’ basic psychological needs (Blanchard et al., 2009). Coaches’ transformational leadership can effectively enhance team cohesion (Price and Weiss, 2013) and promote athletes’ generation of self-sacrificing spirit. Thus, having a sense of team belonging improves athletes’ well-being (Cronin et al., 2015). Some studies have also shown that coaches’ transformational leadership can improve athletes’ satisfaction in collective sports (Álvarez et al., 2010; Saybani et al., 2013) in terms of satisfying athletes’ basic psychological needs and well-being (Kao and Tsai, 2016). The current study’s results indicate that the improvement of athletes’ satisfaction brought about by coaches’ transformational leadership can generate improved basic psychological needs satisfaction and well-being. Therefore, irrespective of the cohesion or satisfaction perspectives, this study’s results can explain the mediating role of basic psychological needs in the relationship between coaches’ transformational leadership and athletes’ well-being. This finding is consistent with Sterling and Tafvelin (2014), who showed that basic psychological needs mediated the relationship between coaches’ transformational leadership and athletes’ well-being, thus laying the foundation for the current study. However, Sterling and Tafvelin (2014) asserted that their research results should be further verified using different sports and cultural backgrounds, as their research measured coaches’ transformational leadership using the Transformational Teaching Questionnaire (TTQ), which was developed by Beauchamp in the physical education context. Thus, the questionnaire’s fitting degree is not ideal for the competitive sports context (Sterling and Tafvelin, 2014). Therefore, the current study uses the DTLI scale in the competitive sports context to further study football, basketball, and volleyball athletes. It explores the moderating role of gender in the relationship between coaches’ transformational leadership and athletes’ well-being to advance the understanding of the impact of coaches’ transformational leadership.

### 4.3. Moderating role of gender

This study reveals that perceived transformational leadership from coaches positively affects athletes’ basic psychological needs and improves their well-being. Female athletes have a higher basic psychological needs score than male athletes, which may be attributed to the following. First, girls’ psychological maturity and personality develop more rapidly than boys during adolescence (Cohn, 1991). Therefore, female athletes may be more mature than male athletes in dealing with interpersonal relationships. These interpersonal relationships (especially with coaches) could result in higher basic psychological needs satisfaction. Second, the psychological research has shown that females with masculine traits have a significantly higher sense of well-being than females with no such traits (Cai et al., 2008). Under the long-term guiding ideology and training mode of “technique and tactics masculinity,” Chinese female basketball, volleyball, and football athletes are in constant conflict against each other and display masculine traits (Gao, 1990; Zhang, 2018). However, while these traits have no negative impact on female athletes, they could enhance their sports performance and make them happier (Zhang and Yao, 2010; Bormann et al., 2016). Third, coaches’ leadership styles can affect athletes’ psychological adaptability; in this process, female athletes show higher adaptability than male athletes (Chen, 2003). Finally, female athletes show lower psychological blocking and higher psychological satisfaction than male athletes (Li, 2019). The ultimate goal of coaches’ transformational leadership is to expand the needs and desires of athletes at a higher level by making them realize the significance and responsibility of the tasks that they undertake (Charbonneau et al., 2001). Therefore, the differences in psychological adaptation, psychological blocking, and psychological satisfaction between male and female athletes leads to different levels of well-being.

### 4.4. Practical implications

Coaches should show more transformational leadership to meet athletes’ basic psychological needs and improve their well-being, as follows. First, during daily training and management, coaches should play an exemplary role and become the role models that athletes are eager to imitate through their words and deeds. Second, coaches should pay attention to the individualized care of athletes and fully stimulate the strengths and intelligence of each so that they feel a certain degree of autonomy and as though they are an indispensable part of the team. When athletes perceive their basic psychological needs to be satisfied in terms of autonomy, competence, and sense of belonging, their subjective well-being will improve; simultaneously, the athletes’ sports performance will also improve. Since females are more sensitive to interpersonal relationships, their interpersonal networking skills are more stable. As sports teams are relatively closed environments, the characteristics of the interpersonal relationships within sports teams often have a more significant impact on female athletes’ psychological needs and well-being. This places higher requirements on coaches’ transformational leadership; coaches must show more transformational leadership to meet female athletes’ basic psychological needs and improve their well-being. Male athletes are more likely to have lower improvements in their well-being than female athletes when their basic psychological needs are met. Therefore, coaches and managers of sports teams should not only focus on meeting male athletes’ basic psychological needs, but should also actively pay attention to the influence of other factors on male athletes’ well-being. This is an essential direction for this study’s future research.

### 4.5. Limitations and directions for future research

This study has the following limitations. First, it used cross-sectional self-reported data from athletes, with no corresponding coaching evaluation scale. However, this study maximized the sample size to reduce the impact of common method bias. This study could collect data from multiple perspectives in the future. Second, this study uses the Chinese context, so the future studies could verify whether the conclusions are generalizable to other contexts. Third, as the sample participants were aged between 15–19 years, is it possible that their limited “life experience” (especially outside of sports) may have been a significant factor in their relationships with their coaches. It would be difficult to find an equivalent sample in the corporate world. Could it be that young people are more or less susceptible to the influence of their coaches/leaders? This study may utilize a similar approach for adults or other organizations in the future to continue to verify the conclusions drawn herein.

## 5. Conclusion

Perceived transformational leadership from coaches significantly and positively affects athletes’ well-being. When coaches show more transformational leadership, athletes’ well-being increases. Athletes’ basic psychological needs mediate the relationship between perceived transformational leadership from coaches and athletes’ well-being. Further, perceived transformational leadership from coaches has a direct impact on athletes’ well-being and an indirect impact on their basic psychological needs being met. The mediating model reveals that gender moderates the relationship between coaches’ transformational leadership and athletes’ well-being, which is mediated by athletes’ basic psychological needs. Specifically, after athletes’ basic psychological needs are satisfied, the well-being of female athletes improves more than that of male athletes.

## Data availability statement

The raw data supporting the conclusions of this article will be made available by the authors, without undue reservation.

## Ethics statement

The studies involving human participants were reviewed and approved by the Academic Ethics Committee of Shandong First Medical University (No. R202202220014). All adult participants provided their informed consent. Written informed consent to participate in this study was provided by the participants’ legal guardian/next of kin.

## Author contributions

WL designed the research and completed the manuscript. SY designed the research with WL and proposed the discussion. WW revised and checked the whole manuscript. All authors contributed to the article and approved the submitted version.

## Funding

SY received funding support from the National Social Science Fund of China (18BTY116).

## Conflict of interest

The authors declare that the research was conducted in the absence of any commercial or financial relationships that could be construed as a potential conflict of interest.

## Publisher’s note

All claims expressed in this article are solely those of the authors and do not necessarily represent those of their affiliated organizations, or those of the publisher, the editors and the reviewers. Any product that may be evaluated in this article, or claim that may be made by its manufacturer, is not guaranteed or endorsed by the publisher.
